# Effectiveness of Real-Time CT/MRI-US Fusion Imaging in Thermal Ablation of Ultrasonographically Inconspicuous Hepatocellular Carcinoma

**DOI:** 10.1007/s00270-025-04302-5

**Published:** 2026-01-08

**Authors:** G. C. M. van Erp, C. A. M. Verhagen, T. J. Koolstra, J. J. van Duijn-de Vreugd, P. Hendriks, M. E. Tushuizen, C. S. P. van Rijswijk, A. R. van Erkel, R. W. van der Meer, M. J. Coenraad, J. Dijkstra, M. C. Burgmans

**Affiliations:** 1 Department of Radiology, Leiden University Medical Center, Leiden, The Netherlands; 2 Department of Gastroenterology and Hepatology, Leiden University Medical Center, Leiden, The Netherlands

**Keywords:** Thermal ablation, Hepatocellular carcinoma, Image guidance, Fusion imaging

## Abstract

**Purpose:**

This study aims to compare local recurrence-free survival (LRFS) in patients with de novo HCC treated with thermal ablation (TA) using real-time CT/MRI-US fusion imaging (FI) or ultrasound (US) for needle placement.

**Materials and Methods:**

This single-center retrospective cohort study included patients with de novo HCC who underwent percutaneous TA between January 2013 and December 2021. US was the preferred image guidance modality for ultrasonographically conspicuous lesions; however, for inconspicuous lesions, FI (US-CT or US-MRI) was used for needle placement. Propensity score matching (PSM) with a 1:1 ratio was applied to balance baseline variables between the US- and FI-guided groups. LRFS, disease-free survival (DFS), and overall survival (OS) were compared before and after matching using the log-rank test. Univariate analyses using Cox regression were used to identify prognostic factors for LRFS.

**Results:**

A total of 117 patients with 157 lesions were ablated using US and FI needle guidance in 100 and 57 tumors, respectively. PSM yielded 40 tumors in both groups. The 1-year LRFS rates were similar across the groups before and after matching (US: 0.82, FI: 0.94 (*p* = 0.07) and US: 0.87, FI: 0.91 (*p* = 0.20), respectively). Univariate analysis revealed that only tumor size was a predictive factor for LRFS. Before and after matching, the DFS and OS did not significantly differ between the groups (*p* > 0.05).

**Conclusion:**

FI-guided needle placement facilitates effective targeting of HCC lesions that are ultrasonographically inconspicuous, yielding LRFS outcomes comparable to those achieved with US guidance for ultrasonographically conspicuous lesions.

*Level of Evidence 3b*, Retrospective Cohort Study.

**Graphical Abstract:**

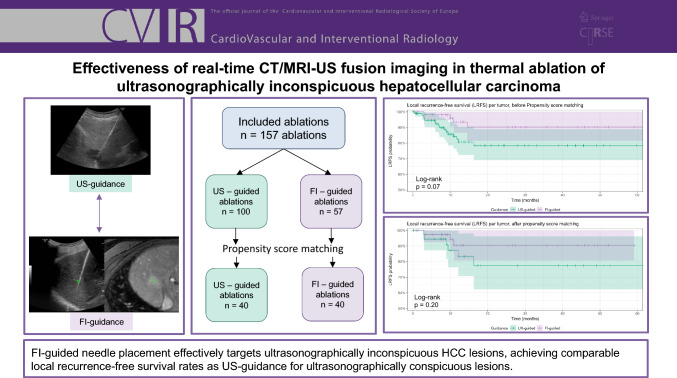

## Introduction

Thermal ablation (TA) is a first-line treatment option for very early and early stage hepatocellular carcinoma (HCC), according to the Barcelona Clinic Liver Cancer (BCLC) staging system [1]. Moreover, it is a suitable treatment as a bridge to liver transplantation [2, 3].

Image guidance is a crucial part of TA, as suboptimal needle placement can lead to incomplete tumor coverage, increasing the risk of residual tumor or local tumor progression (LTP) [4]. B-mode ultrasound (US) is the most widely employed modality for image guidance as it is readily available, allows real-time imaging during needle placement, is time efficient, and is associated with relatively low costs [5]. However, 23.5–31.2% of HCC lesions are inconspicuous on US [6, 7].

Fusion imaging (FI) offers an alternative that combines the real-time feedback of US with the high spatial resolution of cross-sectional imaging modalities such as contrast-enhanced CT (CECT) or MRI. Using electromagnetic tracking, the position and orientation of the US transducer are determined, and the real-time US images are co-registered with the diagnostic scans using anatomical landmarks. This real-time overlay of intraprocedural US with CT or MRI enables visualization of 31.7–45.0% of US-inconspicuous lesion, with comparable ablation complication rates of 9.4% and 0.7–1.9% for minor and major complications, respectively [7–11]. Modern advancements in image registration algorithms and software have made this fusion process more reliable and widely applicable [12].

Although the technical effectiveness and safety of FI have been well established, there is still a lack of large-scale studies comparing oncological outcomes between US- and FI-guided ablations. Therefore, the aim of this study was to compare local recurrence-free survival (LRFS) in patients with de novo HCC treated with TA guided by real-time FI and US for needle placement.

## Materials and Methods

### Patients

This retrospective single-center cohort study included patients with de novo HCC who underwent US- or FI-guided percutaneous thermal liver ablation between January 1, 2013, and December 31, 2021, at the Leiden University Medical Center, a tertiary referral center in the Netherlands. HCC was diagnosed based on imaging or histopathological findings, in accordance with the European Association for the Study of the Liver (EASL) criteria [13]. The decision to perform TA was made by a multidisciplinary tumor board, and treatment was performed with curative intent or as bridge to transplant. Patients were excluded if a) they received combination therapy, i.e., TA combined with transarterial chemoembolization or transarterial radioembolization, b) documentation of the image modality used for guidance was lacking, or c) histopathological assessment revealed a diagnosis other than HCC. The study was approved by the Institutional Review Board, and the requirement for informed consent was waived. Patients’ electronic medical records were retrospectively reviewed. A subset of patients was included in the prospective monocenter IAMCOMPLETE study (14 of 117) and prospective multicenter PROMETHEUS study (4 of 117) [14, 15].

### Thermal Ablation Procedure

**Fig. 1:**
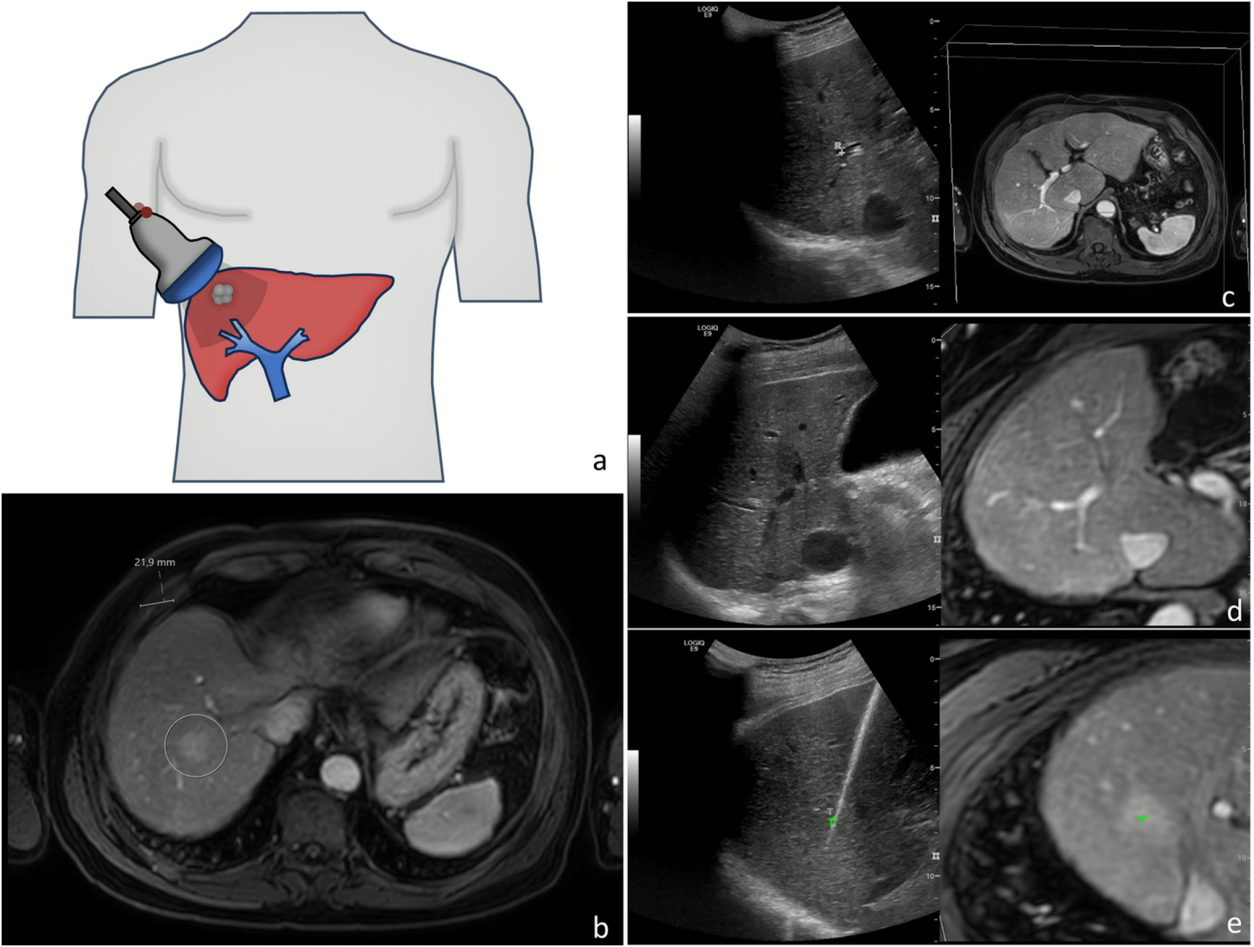
Illustration and clinical example of fusion imaging-guided targeted ablation for an ultrasonographically inconspicuous lesion. (**a**) The ultrasound probe is equipped with two magnetic sensors (red) tracked by an electromagnetic transmitter positioned near the patient. (**b**) Diagnostic MRI shows a hypervascular lesion (22 mm) in segment 7. (**c**) Plane registration is initiated by selecting corresponding planes on MRI and real-time ultrasound. (**d**) Registration is refined using anatomical landmarks. (**e**) A target point (green marker, T) is chosen based on the MRI and projected on the real-time US to guide needle positioning. Microwave ablation (Emprint, 100 W for 10 min) was performed. Post-ablation CT confirms accurate localization of the ablation zone

All TA procedures were performed by four board-certified interventional radiologists. All patients were treated under general anesthesia using either radiofrequency ablation (RFA) or microwave ablation (MWA). RFA procedures were performed with Cool-tip ablation system (Medtronic, Minneapolis, USA) using single or multiple electrodes. Two different single antenna MWA systems were used throughout the study period: Amica (HS Hospital Service, Rome, Italy), and Emprint (HP) (Medtronic, Minneapolis, USA). US-guided needle placement was used for ultrasonographically conspicuous lesions, while FI (LOGIQ E9/E10, General Electric, Boston, USA) was employed for ultrasonographically inconspicuous lesions. The fusion imaging systems combined electromagnetic tracking with image registration. A transmitter was placed near the patient, and two small magnetic sensors were attached to a bracket connected to the US transducer. Using a position-sensing unit, 3D spatial registration of the transducer within the magnetic field was achieved. Diagnostic images in DICOM format were uploaded to the ultrasound machine. Initial plane registration was performed by identifying the portal vein bifurcation, followed by refinement using at least three anatomical landmarks in close proximity to the tumor, such as vessel bifurcations, cysts, and/or calcifications. A target point was then placed on the tumor in the diagnostic scan and projected onto the real-time US to guide tumor targeting (Fig. 1).

Ablation settings were chosen with the objective to obtain a complete tumor ablation with a minimum safety margin of 5 mm in all directions. Hydrodissection was used when adjacent structures were at risk of thermal injury. Upon completion of the ablation, the needle was removed using tract ablation.

Technical success was defined as complete coverage of the tumor by the ablation zone, with no evidence of residual enhancing tumor tissue. CECT was acquired immediately after the ablation procedure, while the patient was still under general anesthesia, to determine technical success using visual assessment supported by two-dimensional measurements. In case of residual tumor enhancement, immediate reablation was carried out. To minimize the risk of contrast-induced nephropathy, intraprocedural CECT was limited to two scans per procedure. If reablation was performed after the acquisition of the second scan, technical success was confirmed with a post-procedural CECT the day following the ablation procedure.

### Follow-up and Outcome Measures

During the first year following TA, patients were monitored every 3 to 4 months and every 6 months thereafter, with follow-up including liver MRI and/or CECT of the chest and abdomen, along with blood tests. The primary outcome measure was LRFS. Secondary outcome measures included rates of residual tumor, defined as tumor presence within or at the ablation zone edge on first follow-up imaging, and LTP, defined as tumor appearance within or at the edge of the ablation zone (< 5 mm) after an initial disease-free follow-up scan. Local recurrence (LR) was defined as either residual tumor or LTP. LRFS was calculated as the time interval between TA and the occurrence of LR. Disease-free survival (DFS) was defined as the time interval between TA and disease recurrence, including LR, intrahepatic recurrence, or distant metastasis. Overall survival (OS) was defined as the time interval between TA and death. Censoring occurred at the earliest of one of the following events: liver transplantation, untreatable disease progression, lost to follow-up, or death. Procedure-related complications were registered according to the modified Cardiovascular and Interventional Radiological Society of Europe (CIRSE) classification system [16].

### Statistical Analysis

Statistical analysis was performed using R Statistical Software (version 4.4.0). Continuous variables were reported as mean and standard deviation (SD) or median with interquartile range (IQR), as appropriate. Categorical variables were presented as counts and percentages. The Shapiro–Wilk test was used to assess the normality of continuous variables. Differences between the two image guidance groups were assessed using a t test or a Mann–Whitney U test as appropriate for continuous variables and the Pearson’s chi-squared test or Fisher’s exact for categorical variables. To minimize the effect of potential confounders on selection bias, propensity score matching (PSM) was performed on lesion level. Propensity scores were estimated using logistic regression including the covariates age, presence of cirrhosis, BCLC stage, tumor size, ablation modality, and tumor location. One-to-one nearest neighbor matching was conducted with a caliper distance set to 0.1.

Kaplan–Meier survival analysis with the log-rank test was used to compare 1- and 3-year LRFS between FI-guided and US-guided procedures. DFS and OS were also evaluated using Kaplan–Meier curves. All survival analyses were performed for the matched and unmatched cohorts. Patients in whom both image guidance modalities were used for targeting multiple tumors were excluded from DFS and OS analyses. The residual tumor rates and LTP rates for different guidance techniques were compared using Pearson’s chi-squared test.

Univariate Cox proportional hazards regression was used to identify prognostic factors for LRFS. In the regression analysis, a cluster term was included to correct for within-patient correlation in patients with multiple tumors. A *p* value < 0.05 was considered statistically significant.

## Results

### Patient Characteristics

**Fig. 2:**
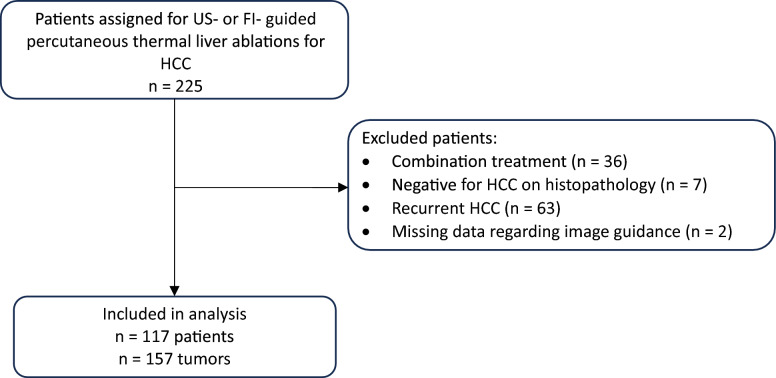
Flowchart of the study population

During the study period, a total of 117 patients with 157 de novo HCC lesions met the inclusion criteria and were included in the analysis (Fig. 2). Baseline patient characteristics are summarized in Table 1. Of the 157 lesions, 100 were ablated under B-mode US guidance and 57 lesions using FI.

**Table 1:** Patient characteristics

	Unmatched cohort (N = 117)	Matched cohort (N = 66)
*Sex*		
Male	95 (81.2%)	54 (81.8%)
Female	22 (18.8%)	12 (18.2%)
*Age (years)*		
Mean (SD)	65 (± 9.2)	67 (± 8.3)
BMI (kg/m^3^)		
Median [IQR]	26 [24, 31]	26 [24, 31]
*Presence of cirrhosis*		
Yes	111 (94.9%)	62 (93.9%)
No	6 (5.1%)	4 (6.1%)
*Child–Pugh class*		
A	73 (65.8%)	40 (64.5%)
B	30 (27.0%)	17 (27.4%)
C	7 (6.3%)	5 (8.1%)
Missing	1 (0.9%)	0 (0%)
*BCLC stage*		
Very early stage	25 (21.4%)	12 (18.2%)
Early stage	76 (65.0%)	44 (66.7%)
Intermediate stage	8 (6.8%)	5 (7.6%)
Advanced stage^a^	0 (0%)	0 (0%)
Terminal stage^a^	7 (6.0%)	5 (7.6%)
Missing	1 (0.9%)	0 (0%)
*Number of lesions*		
1	78 (66.7%)	40 (60.6%)
2	29 (24.8%)	17 (25.8%)
3	8 (6.8%)	7 (10.6%)
4	2 (1.7%)	2 (3.0%)

^a^Bridging to liver transplantation

BMI, Body Mass Index, SD, standard deviation, BCLC, Barcelona Clinic Liver Cancer

Tumor and treatment characteristics are shown in Table 2. Tumor size differed significantly between groups, with a median tumor size of 20 mm (16–25 mm) in the US group and 15 mm (12–22 mm) in the FI group (*p* = 0.001). The use of MWA also differed significantly between groups (*p* < 0.001), being more frequently used in FI-guided ablations compared with US-guided ablations (45.6% vs 14.0%). PSM retained 66 patients and 80 lesions (n = 40 in both groups). Baseline, tumor, and treatment characteristics were well balanced between the matched cohorts (Tables 1 and 2).

**Table 2:** Tumor and procedure characteristics

	Unmatched cohort (N = 157)			Matched cohort (N= 80)		
	US-guided (N = 100)	FI-guided (N = 57)	*p* value	US-guided (N = 40)	FI-guided (N = 40)	*p* value
*Size (mm)*						
Median [IQR]	20 [16, 25]	15 [12, 22]	0.00149	20 [14, 23]	16 [13, 24]	0.923
*Subcapsular location*						
Yes	36 (36.0%)	22 (38.6%)	0.879	15 (37.5%)	13 (32.5%)	0.815
No	64 (64.0%)	35 (61.4%)		25 (62.5%)	27 (67.5%)	
*Tumor location*						
Left	37 (37.0%)	13 (22.8%)	0.0974	14 (35.0%)	12 (30.0%)	0.811
Right	63 (63.0%)	44 (77.2%)		26 (65.0%)	28 (70.0%)	
*Ablation modality*						
MWA	14 (14.0%)	26 (45.6%)	< 0.001	13 (32.5%)	12 (30.0%)	1
RFA	86 (86.0%)	31 (54.4%)		27 (67.5%)	28 (70.0%)	
*Hydrodissection*						
Yes	13 (13.0%)	3 (5.3%)	0.172	3 (7.5%)	2 (5.0%)	1
No	87 (87.0%)	54 (94.7%)		37 (92.5%)	38 (95.0%)	
*Technical success*^*a*^						
Yes	100 (100%)	56 (98.2%)	0.363	40 (100%)	39 (97.5%)	1
No	0 (0%)	0 (0%)		0 (0%)	0 (0%)	
Unknown	0 (0%)	1 (1.8%)		0 (0%)	1 (2.5%)	

^a^Unknown due to maximum contrast dose (n = 1)

FI, Fusion imaging; US, Ultrasound; MWA, Microwave ablation; and RFA, Radiofrequency ablation

### Local Recurrence-Free Survival

**Fig. 3:**
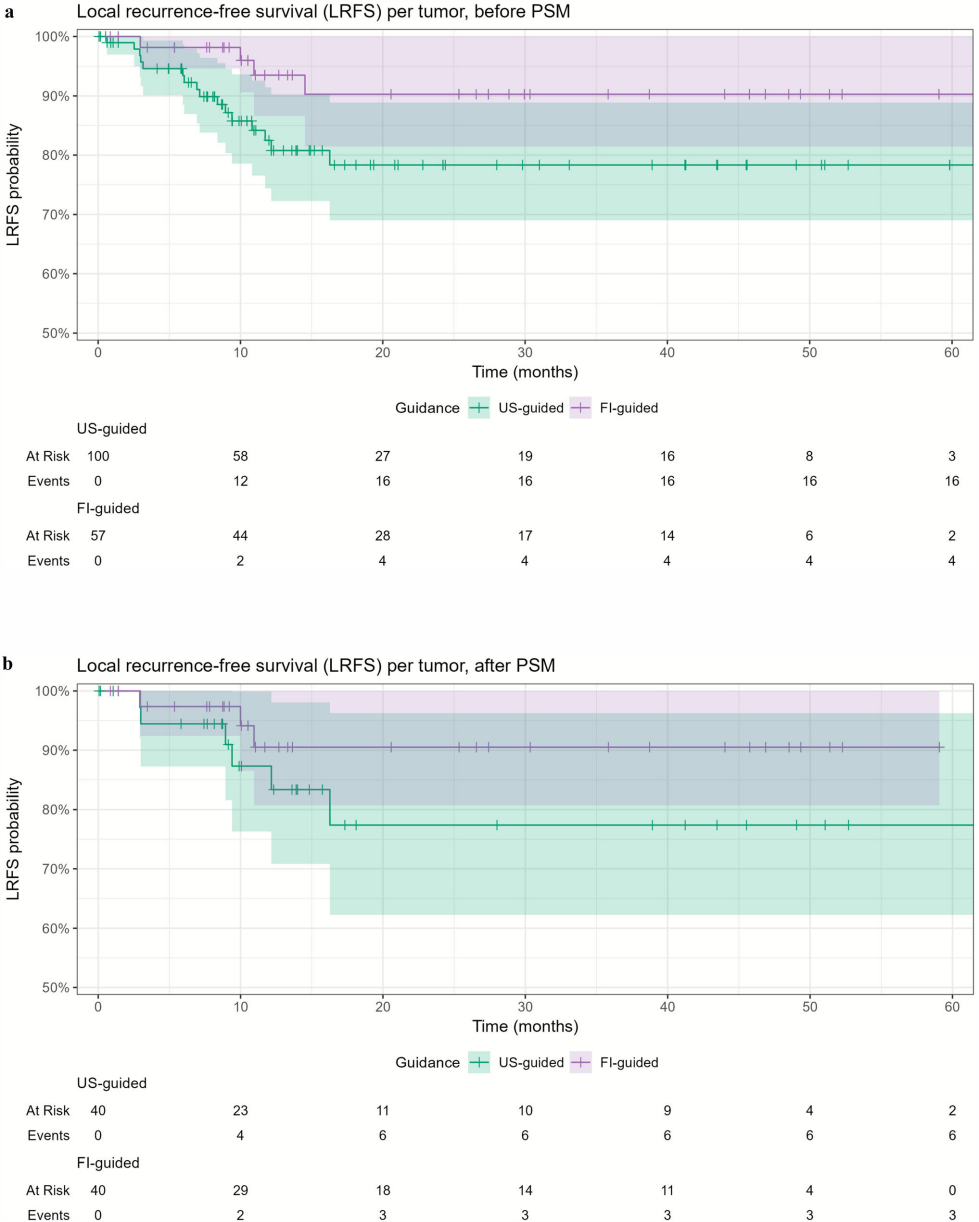
Local recurrence-free survival (LRFS) for US-guided and FI-guided ablations (**a**) before propensity score matching (PSM) and (**b**) after PSM

LRFS did not differ significantly between the groups (*p* = 0.07) (Fig. 3a). The 1- and 3-year LRFS rates were 82.5% and 78.3% for US-guided ablations, and 93.5% and 90.3% for FI-guided ablations, respectively. The mean time to LR was 7.7 months (range 0.6–16.3 months). Univariate Cox regression analysis identified tumor size as a significant variable associated with LRFS (HR: 1.07 (1.02–1.12), *p* = 0.005) (Table 3).

**Table 3:** Univariate Cox regression analysis to identify predictive factors for local recurrence-free survival

	Unmatched cohort (n = 157)	
	HR (95% CI)	p value
Tumor size (mm)	1.07 (1.02–1.12)	0.005
*Image guidance*		
US-guided	Reference	
FI-guided	0.37 (0.13–1.04)	0.06
*Ablation modality*		
MWA	Reference	
RFA	0.72 (0.30–1.71)	0.45
*Tumor location*		
Left	Reference	
Right	0.83 (0.32–2.14)	0.99
*Cirrhosis*		
Yes	Reference	
No	0.62 (0.10–4.05)	0.62

HR, Hazard ratio, CI, Confidence interval, US, Ultrasound, FI, Fusion imaging, MWA, Microwave ablation, RFA, Radiofrequency ablation

After PSM, there was no significant difference between LRFS between the groups (*p* = 0.20) (Fig. 3b). The 1- and 3-year LRFS was 87.3% and 77.4% for US-guided ablations, and 90.5% and 90.5% for FI-guided ablations, respectively.

### Residual Tumors and Local Tumor Progression

Before matching, residual tumor was observed in 6 tumors: 5 after US-guided ablation (5.0%) and 1 after FI-guided ablation (1.8%) (*p* = 0.32). LTP occurred in 14 tumors: 11 after US-guided ablation (11.0%) and 3 after FI-guided ablation (5.3%) (*p* = 0.25).

### Complications

Procedure-related complications occurred in 8.1% (6/74) of patients in the US-guided group and 2.9% (1/35) in the FI-guided group (*p* = 0.313). In the US-guided group, three peri-procedural hemorrhages were observed and were managed conservatively (n = 2) or with embolization (n = 1) (Grade Ia). One patient developed post-procedural encephalopathy three days after ablation, requiring hospitalization (Grade IIIa). Two patients developed segment 3 hepatic infarctions (Grade IIIb). In the FI-guided group, one pseudoaneurysm within the ablation zone required embolization (Grade IIIa) (Table 4).

**Table 4:** Complications graded according to the CIRSE classification system

Grade	US-guided (N = 74)	FI-guided (N = 35)	Combined guidance (N = 8)
None	68 (91.9%)	34 (97.1%)	8 (100%)
Grade 1a	3 (4.1%)	0 (0%)	0 (0%)
Grade 1b	0 (0%)	0 (0%)	0 (0%)
Grade 2	0 (0%)	0 (0%)	0 (0%)
Grade 3a	1 (1.4%)	1 (2.9%)	0 (0%)
Grade 3b	2 (2.7%)	0 (0%)	0 (0%)
Grade 4	0 (0%)	0 (0%)	0 (0%)
Grade 5	0 (0%)	0 (0%)	0 (0%)

### Disease-Free Survival

**Fig. 4:**
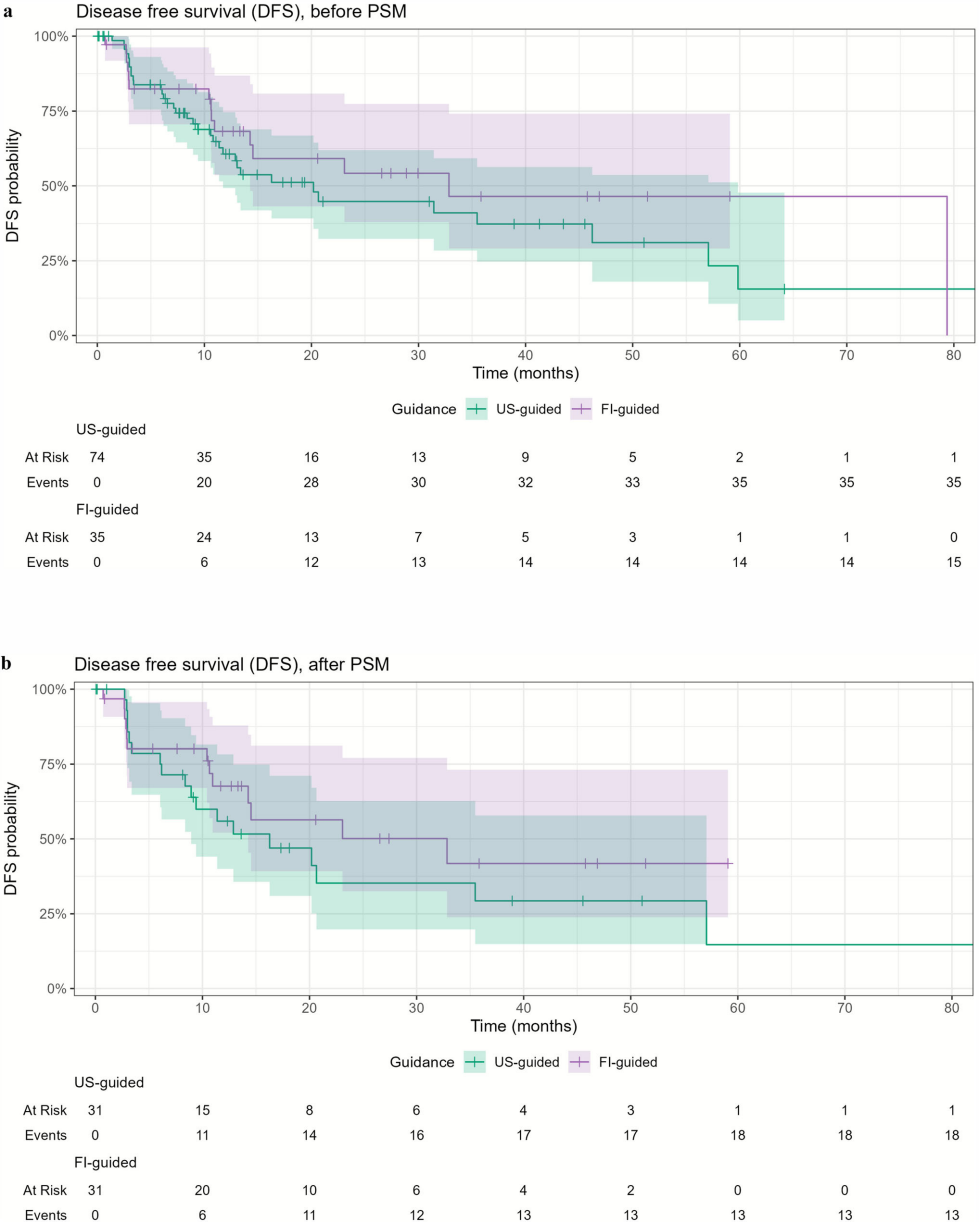
Disease-free survival (DFS) for US-guided and FI-guided ablations (**a**) before propensity score matching (PSM) and (**b**) after PSM

The 1- and 3-year DFS rates were 60.6% and 37.3%, for the US-guided group, and 68.2% and 46.5% for the FI-guided group (p = 0.40) (Fig. 4a). Eight patients presented with extrahepatic metastases involving the bones (n = 2), lungs (n = 3), lungs and brain (n = 1), lymph nodes, bones, and adrenal gland (n = 1), and lungs, lymph nodes, bones, and adrenal gland (n = 1). After matching, the 1- and 3-year DFS rates were 55.9% and 29.3% for the US-guided group, and 67.6% and 41.7% for the FI-guided group, respectively (*p* = 0.30) (Fig. 4b).

### Overall Survival

**Fig. 5:**
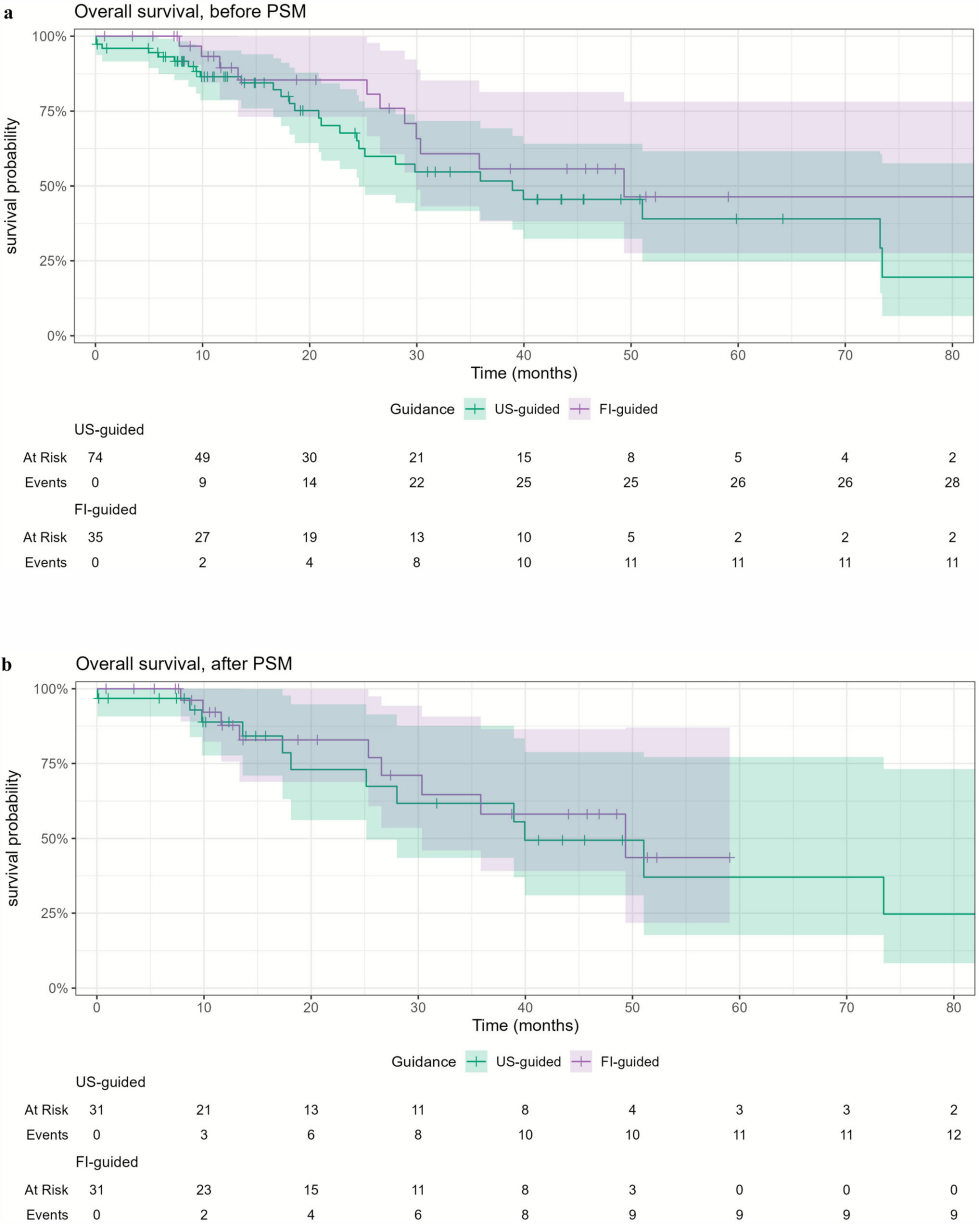
Overall survival for US-guided and FI-guided ablations (**a**) before propensity score matching (PSM) and (**b**) after PSM

The median follow-up duration of the entire cohort was 15.7 months (IQR: 8.7–35.9 months). The 1-, 3-, and 5-year OS rates were 86.5%, 51.6%, and 39.0% for the US-guided group, and 89.5%, 55.7%, and 46.4% for the FI-guided group (*p* = 0.20) (Fig. 5a). Among all patients, 43 died, 42 received a liver transplant, 13 were lost to follow-up, and 7 had untreatable disease progression. Three patients died within 30 days following TA, of whom two had a cardiac arrest and one died of a septic shock following an esophageal varices bleeding. None of these events were directly related to TA.

After matching, the 1- and 3-year OS rates were 88.9% and 61.7% for the US-guided group, and 87.8% and 58.1% for the FI-guided group, respectively (*p* = 0.70) (Fig. 5b). Five-year OS could not be assessed due to the limited number of patients with long-term follow-up in the FI-guided group after matching.

## Discussion

This retrospective single-center study shows that LRFS rates following FI-guided ablations for ultrasonographically inconspicuous de novo HCC lesions are comparable to LRFS rates after US-guided ablations for ultrasonographically conspicuous lesions. The only significant predictive factor for LRFS was tumor size. Residual tumor and LTP rates were lower in the FI group, but this difference was not statistically significant. No significant differences in DFS and OS were found between the two image guidance techniques.

Our study findings are in line with previous studies on FI-guided liver tumor ablations. A prospective study by Ahn et al. found a similar LTP rate of 7.2% in 236 HCC lesions treated with FI-guided TA using RFA [7]. Similarly, Han et al. conducted a prospective study reporting 1- and 3-year LTP rates of 8.6% and 15.2%, respectively, further supporting the effectiveness of FI-guided TA [10]. Other studies compared contrast-enhanced ultrasound (CEUS) FI to (CE)US guidance alone, highlighting its role in improving technique efficacy and fusion accuracy [17–19]. A randomized controlled trial comparing CEUS, CT/MR-CEUS FI, and three-dimensional US-CEUS FI for HCC ablations found no significant difference in LTP rates among the three targeting modalities with 2-year LTP rates of 19.9%, 12.2%, and 9.0% (*p* = 0.105), respectively [19].

FI facilitates successful ablation of US-inconspicuous HCC in different ways. First, when a lesion is inconspicuous on US, a target point identified on the cross-sectional images is translated onto the real-time US to guide needle placement. Second, FI improves spatial orientation and helps direct attention to the region of interest, potentially transforming an inconspicuous lesion into a recognizable one, thereby enhancing its conspicuity during US targeting. Third, FI aids in differentiating true HCC lesions from regenerative nodules and pseudolesions, with reported rates ranging from 7.7% to 16.7%, thereby reducing the risk of mistargeting [9, 20]. Given its ease of use and availability, FI can be readily employed in case of uncertainty.

However, the precision of FI depends on the accuracy of the fusion of the CT or MRI cross-sectional images with the real-time US images. This registration requires expertise and may be prone to errors due to factors such as variations in respiration, patient positioning, and limited visibility of anatomical landmarks [10, 12, 17, 21]. Hakime et al. evaluated the spatial accuracy of CT-US FI in patients and found a spatial fusion accuracy of 11.53 ± 8.38 mm [12]. This accuracy significantly improved when an intraprocedural CT scan immediately before TA was acquired and when the treatment was performed with patients under general anesthesia, limiting liver deformation caused by patient positioning and respiration. In case of landmark-based manual registration, the registration accuracy is further influenced by the visibility of anatomical landmarks, which can be enhanced with CEUS [17]. To overcome this limitation of manual registration and to accelerate the registration process, automatic registration based on vessel segmentation or liver surface can also be employed [10, 22]. In this study, fusion accuracy was not assessed. All patients were treated under general anesthesia, which likely improved FI accuracy. However, intraprocedural CECT was not routinely performed during the study period. To enhance the reliability of image fusion, it is recommended to use general anesthesia, patient immobilization, and intraprocedural CECT. Furthermore, advanced ventilation techniques, such as high-frequency jet ventilation, further reduce respiratory liver motion, potentially enhancing registration stability during needle placement [23].

In recent years, advanced techniques have emerged for the ablation of US-inconspicuous lesions, including stereotactic navigation, robotic guidance, Hepatic Arteriography with C-arm CT-Guided Ablation (HEPACAGA), and CT hepatic arteriography-guided liver ablations, with excellent results [24–28]. Hepatic arteriography guidance, however, requires an additional catheter placement, which increases cost, prolongs procedure time, and introduces logistical challenges. Furthermore, most of these techniques require a static liver position during targeting, necessitating high-frequency jet ventilation or temporary apnea. FI offers distinct advantages, including real-time probe position tracking, the absence of radiation exposure, relative time efficiency, flexibility in choosing the needle access plane, and lower cost, making it suitable for centers with limited technical resources. FI also enables the targeting of vanishing hepatic metastases treated with neoadjuvant systemic therapy invisible on intraprocedural CT or MRI scans. Overall, FI provides a solution that is both simple and widely available for ablating US-inconspicuous lesions. We firmly believe FI should be part of the armamentarium of the interventional oncologist as even advanced techniques can fail when lesion visibility on CT- of CBCT-hepatic arteriography is poor or when technical errors occur.

This study has several limitations. First, it is a retrospective, single-center study covering a nine-year period (2013–2021) which introduces potential bias due to evolving treatment protocols. Furthermore, selection bias may have influenced study outcomes despite the PSM. As smaller tumors are more likely to be inconspicuous on US, mean tumor size was statistically significantly lower in the FI group (mean 17 mm) compared to the US group (21 mm for both). Lastly, the small sample size limits the statistical power of the study and may reduce the reliability of the univariate Cox regression. Due to the limited number of events, a multivariate analysis was not conducted.

## Conclusion

In conclusion, FI-guided needle placement is an effective and accessible method to target de novo HCC lesions that are ultrasonographically inconspicuous, yielding LRFS outcomes comparable to those achieved with US guidance for ultrasonographically conspicuous lesions.
